# Improving the Post-polymerization Modification of Bio-Based Itaconate Unsaturated Polyesters: Catalyzing Aza-Michael Additions With Reusable Iodine on Acidic Alumina

**DOI:** 10.3389/fchem.2019.00501

**Published:** 2019-07-15

**Authors:** Oliver B. Moore, Polly-Ann Hanson, James W. Comerford, Alessandro Pellis, Thomas J. Farmer

**Affiliations:** Green Chemistry Centre of Excellence, Department of Chemistry, University of York, York, United Kingdom

**Keywords:** biopolymer, itaconic acid, enzymatic polycondenzation, heterogeneous catalyst, Michael addition, mesaconate

## Abstract

Bio-based platform molecules such as itaconic, fumaric, and muconic acid offer much promise in the formation of sustainable unsaturated polyester resins upon reaction with suitable diols and polyols. The C=C bonds present in these polyester chains allows for post-polymerization modification and such moieties are conventionally utilized in curing processes during the manufacture of coatings. The C=C modification sites can also act as points to add useful pendants which can alter the polymers final properties such as glass transition temperature, biodegradability, hardness, polarity, and strength. A commonly observed modification is the addition of secondary amines via an aza-Michael addition. Conventional procedures for the addition of amines onto itaconate polyesters require reaction times of several days as a result of undesired side reactions, in particular, the formation of the less reactive mesaconate regioisomer. The slow reversion of the mesaconate back to itaconate, followed by subsequent amine addition, is the primary reason for such extended reaction times. Herein we report our efforts toward finding a suitable catalyst for the aza-Michael addition of diethylamine onto a model substrate, dimethyl itaconate, with the aim of being able to add amine onto the itaconate units without excessive regioisomerization to the inactive mesaconate. A catalyst screen showed that iodine on acidic alumina results in an effective, heterogeneous, reusable catalyst for the investigated aza-Michael addition. Extending the study further, itaconate polyester was prepared by *Candida Antartica Lipase B* (CaL-B) via enzymatic polytranesterification and subsequently modified with diethylamine using the iodine on acidic alumina catalyst, dramatically reducing the required length of reaction (>70% addition after 4 h). The approach represents a multidisciplinary example whereby biocatalytic polymerization is combined with chemocatalytic modification of the resultant polyester for the formation of useful bio-based polyesters.

## Introduction

**Figure 1:**
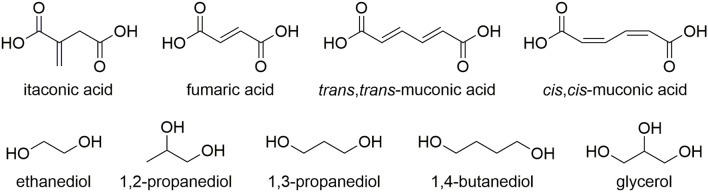
Common bio-derivable unsaturated diesters, diols, and polyols used in the synthesis of unsaturated polyester resins (UPEs).

**Figure 2:**
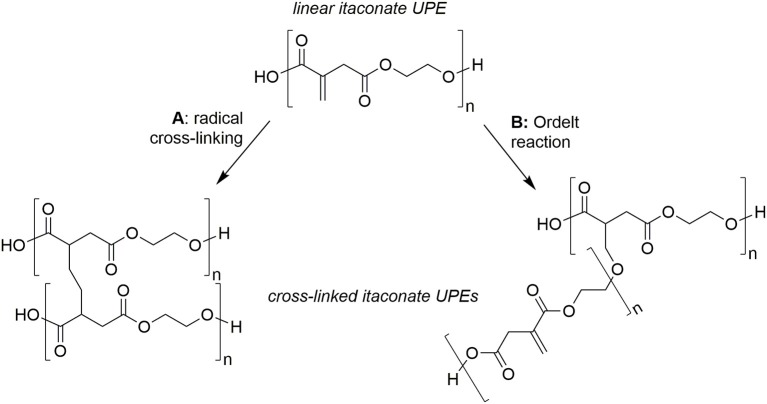
Typical undesired side-reactions of bio-based unsaturated polyesters (UPEs), example for poly(ethylene itaconate). **(A)** Radical induced cross-linking via C=C, **(B)** Ordelt saturation (oxo-Michael addition of –OH end-group onto C=C) inducing crosslinking.

An over-reliance of the chemical industry on non-renewable feedstocks has resulted in an ever growing interest in the utilization of bio-derived platform molecules to substitute petroleum-derived base chemicals as fundamental building-blocks for the synthesis of higher value products (Werpy and Petersen, 2004; Farmer and Mascal, 2014). Due to the sheer volumes produced coupled with enormous diversity of applications, it is not surprising that the field of polymer science has shown particular interest in using platform molecules to sustainably source monomers or monomer precursors (Mathers, 2012; Gandini and Lacerda, 2015; Isikgor and Becer, 2015; Llevot et al., 2016; Zhu et al., 2016). Plastics such as poly(lactic acid) (PLA), poly(butylene succinate) (PBS), and poly(ethylene furanoate) (PEF) demonstrate how polymers with favorable properties can be partly or wholly derived from platform molecules. A more recent trend has been toward the synthesis of functionalizable polymers and in particular, the polymerization of common platform molecules, itaconic acid, muconic acid, and fumaric acid with a range of diols/polyols such as 1,2-ethanediol, 1,2-propandiol, 1,3-propandiol, 1,4-butanediol, and glycerol (Figure 1) and to produce novel, 100% bio-derived unsaturated polyesters (UPEs) (Fonseca et al., 2015, 2017; Robert and Friebel, 2016; Rorrer et al., 2016; Costa et al., 2017; Kumar et al., 2017; Patil et al., 2017; Farmer et al., 2018a). Synthesis of these sustainable UPEs often employs conventional melt polymerization methods using well established metal catalysts (Ti, Al, Sn, and Zn) (Sakuma et al., 2008; Chanda and Ramakrishnan, 2015; Farmer et al., 2015; Winkler et al., 2015; Rowe et al., 2016; Schoon et al., 2017). Although valuable C=C groups present on such monomers offer enhanced functionality to the UPEs, they can also create many issues with the formation of undesired side reactions during the polymerization. In the case of itaconates, fumarates, and muconates typical side reactions include isomerization, radical cross-linking (Figure 2A) and Ordelt saturation (an oxo-Michael addition where an R-OH end-group attacks the conjugated C=C through a β-addition, Figure 2B; Farmer et al., 2015; Schoon et al., 2017).

**Figure 3:**
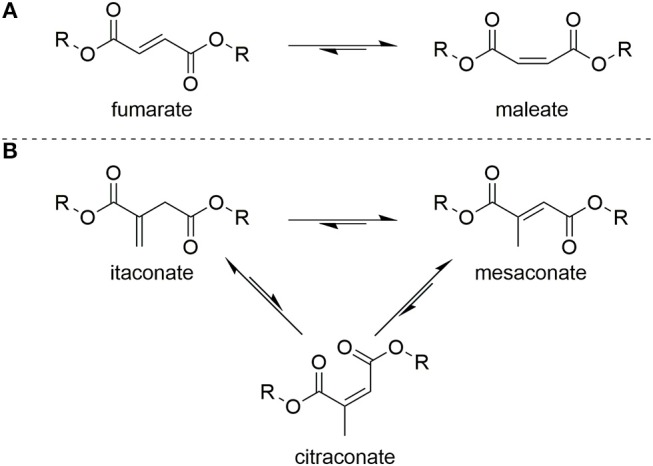
Isomerization between **(A)** fumarate and maleate units in UPEs or **(B)** itaconate and mesaconate/citraconate units in UPEs.

To prevent radical crosslinking during conventional polyesterification of such monomers, scavengers such as quinol (Chanda and Ramakrishnan, 2015) and 4-methoxyphenol are used as quenchers (Schoon et al., 2017). Enzyme catalyzed polytransesterifications have proven somewhat effective in limiting isomerization but achieving high degrees of polymerization and efficient scaling-up these methods had proven elusive (Corici et al., 2015; Pellis et al., 2015). Limiting Ordelt saturation has proven even harder to achieve, most likely due to the fact that Lewis acid catalysts promoting polytransesterification will also increases the ability of the conjugated C=C to act as a Michael acceptor to a hydroxyl end-group. Once extensive crosslinking occurs the resultant UPEs, for example when using itaconates, are typically soft and rubbery and thus are only suitable for applications which do not require inherent strength (Singh et al., 1991; Guo et al., 2011; Wei et al., 2012; Dai et al., 2015a,b,c). Undesirable isomerization of the C=C is also widely reported for UPEs of itaconate, muconate fumarate, and maleate monomers, with the latter two able to interchange between one another (Figure 3A). In the case of itaconate containing polyesters, regioisomerization during polyesterification results in the formation of mesaconate (major) and citraconate (minor) units (Figure 3B). Formation of these regio-isomer units lead to greater complexity in the analysis of the polyesters, whilst also effecting reproducibility of the polymers final thermal and mechanical properties.

**Figure 4:**
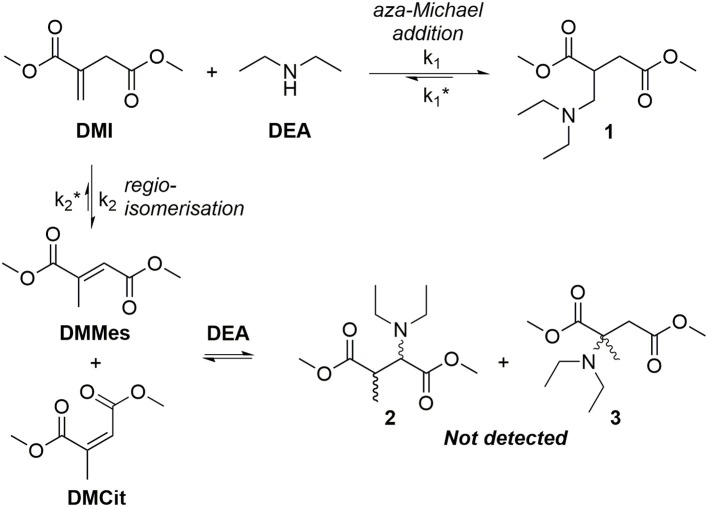
Competitive reaction between either the addition of diethyl amine (DEA) to dimethyl itaconate (DMI) or regioisomerization of DMI to form dimethyl mesaconate (DMMes, dominant isomer) or dimethyl citraconate (DMCit, minor isomer).

Previous research has continually observed regioisomerization of itaconate during polyester synthesis. The extent of this undesired reaction ranges from <10% (relative to itaconate) as described by Teramoto (9%) (Teramoto et al., 2005), Farmer (8%) (Farmer et al., 2015), and Spavojevic (7%) (Panic et al., 2017), increasing up to nearly 60% in the Takasu's protocol using itaconic anhydride as the monomer (Takasu et al., 1999). In the majority of the above examples the typical reaction conditions are elevated temperatures above 160°C, high vacuum to remove excess diol and relatively long reaction times and acidic catalysts or polymer chain ends; these conditions contribute to the promotion of mesaconate formation. The most effective method to avoid underdesired regioismerization during polycondenzation is to carry out the reaction under milder enzyme-catalyzed conditions (Pellis et al., 2019). However, regioisomerization has also been reported to occur during addition of pendants to free unsaturated sites, with a significantly increased proportion of mesaconate being seen for high (>75%) but not complete addition (Clark et al., 2009; Farmer et al., 2016). Pendant addition to itaconate UPEs remains a very desirable pathway as significant alterations to the polymers physical properties can be achieved, whilst the pendants themselves exhibit behavior such as metal chelation. Several recent studies have demonstrated post-polymerization modification (PPM) of bio-based UPEs, allowing these polyester backbones to be altered via facile Michael additions (Fonseca et al., 2015, 2017; Robert and Friebel, 2016; Rorrer et al., 2016; Costa et al., 2017; Kumar et al., 2017; Patil et al., 2017; Farmer et al., 2018a). Additions of thiols, amines and metal-chelating 1,3-dicarbonyls to bio-based UPEs have been recently demonstrated, tailoring the properties of the polyesters to suit a range of applications (Lv et al., 2014; Chanda and Ramakrishnan, 2015; Farmer et al., 2016). For example Hoffmann et al. showed that amine pendant addition to itaconate polyesters can tune the hydrophilicity of resultant gels and be tailored depending on the choice of amine donor (Hoffmann et al., 2015). Amine pendant itaconate polyesters have recently been used to produced temperature switchable materials, with examples of low-temperature depolymerization promoted by primary amine addition (Guarneri et al., 2019) or secondary amine release at elevated temperatures (>190°C) (Pellis et al., 2019). However, in many instances long reaction times for the addition of the pendants are quoted with little or no discussion as to why this is necessary. Lv et al. reported the need for 14–20 h reaction times for the addition of thiols and amines, despite the Michael donors being used in a 15-times molar excess (Lv et al., 2014). Chanda reported 3 day long additions of both thiols and amines to poly(dodecyl itaconate) UPEs (Chanda and Ramakrishnan, 2015), while Winkler published thio-Michael additions that ran overnight using a 5-times molar excess of donor, and requiring 10 mol% hexylamine as a catalyst (Winkler et al., 2015). In a recent study it was shown that during aza-Michael addition of diethyl amine (DEA) onto dimethyl itaconate (DMI, a mimic for the itaconate unit in polyesters) regioisomerization (k_2_) is in direct competition with desired addition (k_1_) (Figure 4; Farmer et al., 2018b). Mesaconate (DMMes) formation was proven to be catalyzed by the amine Michael donor (DEA) but because the DMMes itself does not act as an acceptor (compounds **2** or **3** were not detected, Figure 4) the lengthy reaction times were deemed to be necessary. These long reaction times were found to be a result of the slow reformation of DMI from DMMes (${\text{k}}_{2}^{*}$, Figure 4), with ${\text{k}}_{2}^{*}$ being an order of magnitude slower than k_1_. It was deduced that a suitable catalyst might be able to selectively catalyze the desired aza-Michael addition before extensive undesired regioisomerization occurs, resulting in significantly reduced post-polymerization modification reaction times from several days to just hours. Herein we report a study into finding a suitable catalyst for the aza-Michael addition of DEA onto DMI and an extension of this method to an addition onto poly(1,8-octylene itaconate) UPE.

## Materials and Methods

### Chemicals and Enzymes

Dimethyl itaconate (DMI) and cerium (III) ammonium nitrate (CAN) were purchased from Alfa Aesar. Cerium (IV) ammonium nitrate (CAN) was purchased from FSA. All other chemicals and solvents were purchased from Sigma-Aldrich and used as received if not otherwise specified. For the powdered supports used as received in this study the following information is available from the supplier (Sigma-Aldrich): acidic alumina (19996-6), Brockmann I, 58 Å pore size, 150 mesh; neutral alumina (199974), Brockmann I, 58 Å pore size; basic alumina (199443), Brockmann I, 58 Å pore size; silica K60 (60738), 60 Å pore size, 220–240 mesh. *Candida Antarctica* lipase B (CaLB) immobilized onto methacrylic resin was purchased from Sigma-Aldrich (product code L4777, also known as Novozym 435). The enzyme was dried under vacuum for 96 h at 25**°**C and stored in a desiccator prior to use.

### Preparation of CAN on Silica

Immobilization 1: To a 50 mL round bottomed flask, 1 gram from K60 silica gel was added with 0.2 mmols (109.7 mg) of (NH_4_)Ce(NO_3_)_6_ and 20 mL of MeOH. Equipped with a reflux condenser, the solution was stirred slowly using a magnetic stirrer bar, at room temperature for 2 h. The solvent was then slowly evaporated over an hour under increasing reduced pressure until 10 mbar vacuum was achieved. The resultant bright orange material was then place under high vacuum (<1 mbar) for 2 h and then stored under an inert purge until required. Actual quantities used resulted in a loading of 0.156 mmol g^−1^ CAN on silica.

Immobilization 2: Increased concentrations of physisorbed (NH_4_)Ce(NO_3_)_6_ were prepared as shown above, but using 0.5 mmol of (NH_4_)Ce(NO_3_)_6_ with 1 g of silica giving a loading of 0.403 mmol g^−1^.

### Preparation of Iodine on Alumina or Silica

For the generation of the various forms of iodine (I_2_) on alumina or silica, the standard loading of I_2_ was 0.1 mmol (as I_2_ ≡ 0.2 mmol elemental I) per gram of alumina/silica (Deka and Sarma, 2001). To create this, 0.3804 g of I_2_ was dissolved in 30 mL dichloromethane, before 15 g of the either alumina or silica was added to the reaction mixture. This suspension was then stirred for 30 min, before the excess solvent was removed under reduced pressure at 40°C. The orange/red powder obtained was then left to dry fully overnight before use. For higher loadings the iodine amount was multiplied (2x, 3x, and 4x) as required while all other reagent amounts and procedures were followed as above.

### Enzymatic Polycondenzation for Formation of Poly(1,8-Octylene Itaconate) (POI)

The solventless synthesis procedure was taken from previous literature (Pellis et al., 2018, 2019). Briefly: 6 mmols of dimethyl itaconate and 6 mmols of 1,8-octanediol were accurately weighed into a 25 mL round bottom flask. The mixture was then stirred at 85 **°**C until a homogeneous melt was obtained. Ten percent w w^−1^ calculated on the total amount of the monomers of Novozym 435 was then added to the reaction mixture. The reactions were run for 6 h at 1,013 mbar. A vacuum of 20 mbar was subsequently applied for an additional 18 h maintaining the initial reaction temperature (total reaction time: 24 h). The reaction product was recovered by adding THF to dissolve the POI, the supported CaLB catalyst was removed *via* vaccum-assisted Buchner filtration (Fisherbrand filter paper QL100, 70 mm diameter), and the solvent from the filtrate evaporated under vacuum to yield the POI polymer product. The polymer was characterized (^1^H-NMR spectroscopy in CDCl_3_, Figure S10) without any additional purification steps prior to use.

### Aza-Michael Addition of Diethylamine (DEA) Onto Dimethyl Itaconate (DMI) Using Cerium Catalysts

2.5 mmol of dimethyl itaconate and the selected catalyst (2 mol% Ce relative to DMI) were accurately weighed into an 8 mL flat bottomed sample vial. Twenty millimoles of diethylamine was added to the reaction and stirred at room temperature for 2 or 6 h when kinetic studies were made (taking aliquots at 2 hourly intervals for analysis by ^1^H-NMR spectroscopy, CDCl_3_ solvent). For the recovery of the catalyst the reaction mixture was filtered via vacuum-assisted Buchner filtration (Fisherbrand filter paper QL100, 70 mm diameter), with no solvent washing, the collected catalyst was left to air-dry over night between reuses.

### Aza-Michael Addition of Diethylamine (DEA) Onto Dimethyl Itaconate (DMI) Using Molecular Iodine Catalysts

2.5 mmol of dimethyl itaconate and the selected amount of catalyst (no catalyst, 1.5, 5, 12.5 mol% of I_2_ relative to DMI) were accurately weighed into an 8 mL flat bottomed sample vial. Twenty millimoles of diethylamine was added to the reaction and stirred at room temperature for 2 h. An aliquot of the reaction mixture was dissolved in CDCl_3_ for analysis by ^1^H-NMR spectroscopy.

### Aza-Michael Addition of Diethylamine (DEA) Onto Dimethyl Itaconate (DMI) Supported Iodine Catalysts

2.5 mmol of dimethyl itaconate and the selected catalyst (5%mol w.r.t. I_2_ per mole of DMI = 1.28 g of standard catalyst 1x loading catalyst) were accurately weighed into an 8 mL flat bottomed sample vial. Twenty millimoles of diethylamine was added to the reaction and stirred at room temperature for 2 h. The catalyst was removed by filtration and an aliquot of the filtrate was dissolved in CDCl_3_ for analysis by ^1^H-NMR spectroscopy. For the recovery of the catalyst the reaction mixture was filtered *via* vacuum-assisted Buchner filtration (Fisherbrand filter paper QL100, 70 mm diameter), with no solvent washing, the collected catalyst was left to air-dry over night between reuses. A dry free-flowing powder was obtained from each air drying. For the iodine on alumina loading study the relative quantity of I_2_ (relative to DMI) was increased to 12.5%mol.

### Extended Recovery and Reuse (10 Cycles) of Iodine on Acidic Alumina for the Aza-Michael Addition of Diethylamine (DEA) Onto Dimethyl Itaconate (DMI)

2.5 mmol of dimethyl itaconate and 1.28 g of 0.1 mmol g^−1^ I_2_ on Al_2_O_3_ catalyst (5%mol w.r.t. I_2_ per mole of DMI) were accurately weighed into an 8 mL flat bottomed sample vial. 20 mmol of diethylamine was added to the reaction and stirred at room temperature for 2 h. The catalyst was removed via vacuum-assisted Buchner filtration (Fisherbrand filter paper QL100, 70 mm diameter), with no solvent washing, the collected catalyst was left to air-dry over night between reuses. For each used an aliquot of the filtrate was dissolved in CDCl_3_ for analysis by ^1^H-NMR spectroscopy.

### Aza-Michael Addition of Diethylamine (DEA) Onto Poly(1,8-Octyleneitaconate) (POI)

2.5 mmol of POI (0.6 g, based on constitutional repeat unit of 240.29 g mol^−1^) and 0.64 g of 0.2 mmol g^−1^ I_2_ on Al_2_O_3_ catalyst were accurately weighed into an 8 mL flat bottomed sample vial. Twenty millimoles of diethylamine was added to the vial and stirred for 24 h with aliquots taken at various intervals for analysis by ^1^H-NMR spectroscopy (CDCl_3_ solvent). After 24 h the reaction mixture was filtered to remove the spent catalyst (where used), excess DEA was removed *in vacuo* and the resultant viscous polymer analyzed by ^1^H-NMR (CDCl_3_ solvent) to compare against known previous literature characterization for this material (Pellis et al., 2019). For the non-catalyzed reaction 0.52 g and 0.54 g (duplicate) of polymer was recovered, for the catalyzed reaction 0.33 g and 0.22 g (duplicate) of polymer was recovered.

### Nuclear Magnetic Resonance (NMR) Spectroscopy

^1^H, ^13^C, DEPT and HMQC NMR spectroscopic analysis were performed on a JEOL JNM-ECS400A spectrometer at a frequency of 400 MHz. CDCl_3_ was used as the NMR solvent for itaconate-based polymers and for the aza-Michael addition experiments.

### Gel Permeation Chromatography (GPC)

GPC was carried out using a PSS SDV High set composed of 3 analytical columns (300 × 8 mm, particle diameter 5 μm) of 1,000, 1,000 × 10^5^ and 10^6^ Å pore sizes, plus guard column (Polymer Standards Service GmbH, PSS) installed in a PSS SECcurity SEC system. Elution was with THF at 1 mL min^−1^ with a column temperature of 30 °C and detection by refractive index. Twenty microliter of a ~2 mg mL^−1^ sample in THF, adding a drop of toluene as reference standard, was injected for each measurement and eluted for 50 min. Calibration was carried out in the molecular weight range 370–25,20,000 Da using the ReadyCal polystyrene standards supplied by Sigma Aldrich and referenced to the toluene peak.

### Nitrogen Porosimetry

Nitrogen adsorption measurements were carried out at 77 K using a Micromeritics Tristar Porosimeter. Prior to analysis, the catalyst samples were outgassed at 180°C for 8 h under a flow of nitrogen gas. The specific surface areas were evaluated using the Brunauer–Emmett–Teller (BET) method in the P/P_0_ range 0.05–0.3 (linear range). Pore size distribution curves were calculated using the adsorption branch of the isotherms and the Barrett–Joyner–Halenda (BJH) method, and pore sizes were obtained from the peak positions of the distribution curves.

### Thermogravitmetric Analysis (TGA)

TGA was performed on a PL Thermal Sciences STA 625 thermal analyzer. 10 mg of sample was weighed into an aluminum cup, placed in the furnace with a N_2_ flow of 100 mL min^−1^ and heated from 30 to 625°C at a heating rate of 10°C min^−1^.

## Results and Discussion

Aza-Michael addition onto unsaturated polyesters are typically performed without catalyst, however as previously mentioned this requires extensive reaction times (Blaha et al., 2018; Pellis et al., 2019). Contrastingly, catalysts are often used for aza-Michael additions when synthesizing drug molecules, allowing lower activation energies and thus increased rates of reaction. Example catalysts include boric acid (Chaudhuri et al., 2005), lipases (Dhake et al., 2010), sulfonated zirconia (Reddy et al., 2008), copper(II) acetylacetonate (Kantam et al., 2005), and indium trichloride (Yang et al., 2007) but more commonly observed is the use of lanthanide metal-centered catalysts (e.g., SmI_2_ Reboule et al., 2005 and Yb(OTf)_2_ Jenner, 1995). Such catalysts have been shown to be particularly efficient for promoting aza-Michael additions, their activity assumed to be a result of Lewis acid behavior drawing electron density away from the C=C and making the β-position more susceptible to attack from the nucleophilic amine. Of the lanthanide series, cerium (Ce) is one of the most intriguing as it can readily switch between the (IV) and (III) oxidation states whereas other lanthanides generally tend to only be stable in the (III) oxidation state. Cerium's (IV) state has high oxidizing power (Kilbourn, 1986) and high redox potential meaning that cerium has been widely used as a single electron oxidant, though the salts of cerium as Lewis acids have received somewhat less attention (Sridharan et al., 2007).

### Investigations Into Cerium Ammonium Nitrate as a Catalyst for Aza-Michael Additions

Cerium ammonium nitrate (CAN) is one of the most documented amongst the cerium-based catalysts (Sridharan and Menendez, 2010; So and Leung, 2017). CAN is able to act as a catalyst for a multitude of reactions, including carbon-nitrogen bond forming reactions such as aza-Michael additions (Nair and Deepthi, 2007) and has the benefit of being widely available and relatively cheap. Furthermore, it has also been shown to be an efficient catalyst toward both aliphatic and aromatic amine additions to Michael acceptors, thus offering a broad substrate scope (Duan et al., 2006).

Despite its high activity, recycling of cerium complexes as catalysts has not been well-documented in the literature and whilst a recent review (Molnar and Papp, 2017) on catalyst recycling mentioned two cerium containing complexes, both catalysts had cerium as a minor component. As such, we additionally sought to find an appropriate means of catalyst recovery and reuse in this work.

**Figure 5:**
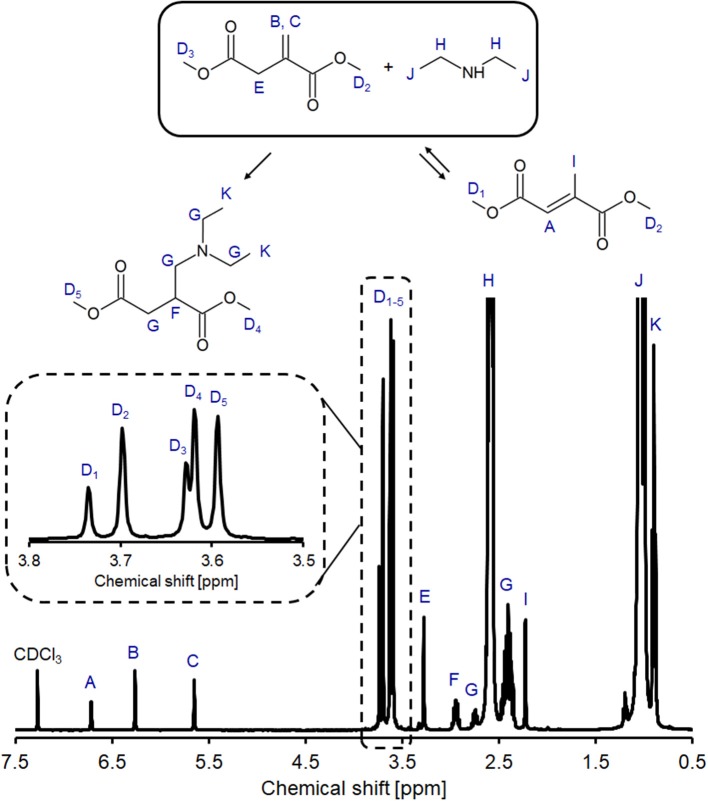
Example ^1^H NMR spectrum of the crude product from aza-Michael reaction between DMI and DEA with 3.8–3.5 ppm expanded region of the spectrum shown. Spectra of the crude reaction mixture was recorded in CDCl_3_ after 2 h of reaction.

In alignment with our previous study into the un-catalyzed addition of amines on polyesters (Farmer et al., 2018b; Pellis et al., 2019), we first elected to study the room temperature addition of DEA using DMI as a model compound (Figure 4) representing the UPRs constitutional repeat unit (CRU). Use of a small model compound also aided analysis and avoided issues of changing reaction viscosity and consequently, effects of mass transfer. ^1^H NMR analysis of the DMI and DEA reaction mixture showed peaks **A** and **I**, confirming the presence of regioisomerization product DMMes (Figure 5). Peak **H** shows C*H*_2_ of the excess unreacted DEA, whilst there is significant overlap between the amine protons and CH_3_ peaks **J**, making these unquantifiable for some samples. Peaks **F** is a complex multiplet as the surrounding H atoms are inequivalent, with a similar observation seen for signal **G**. To calculate the relative amounts of DMI and DMMes, peaks labeled **D** (methyl ester groups, CO_2_CH_3_) were integrated as 6 protons. These peaks are expanded in Figure 5 to demonstrate the detectable difference. Although 6 different environments are possible for the methyl ester groups only five signals are observed; **D2** is a combination of the unsaturated ester of DMI and one of the ester groups of DMMes. Using these signals we were able to quantify the relative molar ratios of DMI (**D2** + **D3**), DMMes (**D1** + **D2**) and desired adduct **1** (**D4** + **D5**) during the course of reaction. The following equation was used (PI = peak integral):

(1) $$\begin{array}{l} \%\;adduct\;1=100\;x\;\frac{\left(PI_{D4}+PI_{D5}\right)}{\left({PI_{D1}+PI_{D2}+PI_{D3}+\;PI}_{D4}+PI_{D5}\right)} \end{array}$$

**Figure 6:**
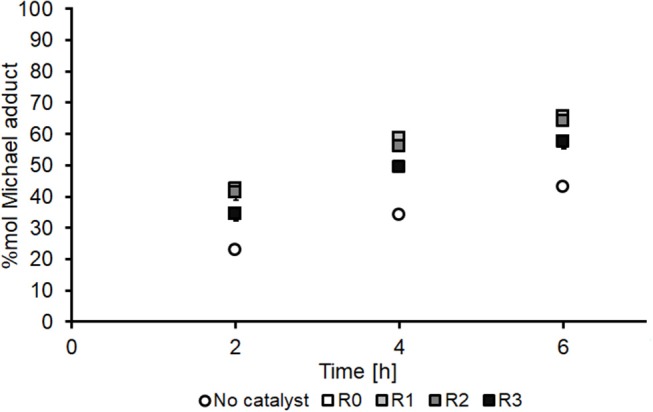
Time course for aza-Michael addition of DEA onto DMI using CAN(IV) as catalyst, showing the first use (R0) and the subsequent three recycles (R1–R3) of the free catalyst and comparison to the no catalyst control. All experiments were performed in duplicates and are shown in the figure as the average of two independent experiments ± the standard deviation. The catalyst was used as received (Sigma-Aldrich) without pre-treatment.

As shown in Figure 6, non-immobilized CAN(IV) (Figure 6, R0, white boxes) noticeably increases the formation of aza-adduct over the studied timescale (2–6 h) relative to the conventional un-catalyzed system (Figure 6, white circles). Interestingly, the CAN(IV) catalyst remained insoluble in the reaction media for the duration of the reaction and was therefore investigated as a potentially recoverable and reusable heterogeneous catalyst (Figure 6, R1–R3, shades of gray boxes). However, CAN's activity was found to steadily decrease upon re-use, particularly upon the third reuse (R3, Figure 6). As such, experiments were conducted to further understand the mechanism of the catalyst deactivation in the DMI + DEA model system, in the hope that it would highlight approaches to both improved adduct yield and enhanced catalyst recycling.

The reduction of Ce(IV) to Ce(III), with regeneration of Ce(IV) by an external oxidant was considered as one possible mechanism of catalysis. If this was the case, it was thought that the addition of a constant supply of oxygen to act as an oxidizing agent for Ce(III), would improve the recyclability allowing Ce(IV) to be constantly regenerated. However, as shown in Figure S1, this was not successful, where the two Ce oxidation states might exhibit similar activity and the addition reaction could be independent of oxidation state. Decrease in catalytic activity of the recycled catalyst was possibly due to the CAN itself deactivating overtime. CAN(IV) has 2 ammonium and 6 nitrate groups, and CAN(III) has 2 ammonium and 5 nitrate groups. In order to test whether the CAN(IV) was losing its nitrate groups, and consequently catalytic ability, the molar equivalent of sodium nitrate was added to the reaction on CAN's 4th recycle in an attempt to regenerate the CAN(IV). However, there appeared to be little benefit in addition of the sodium nitrate to prevent deactivation of either CAN(III) or CAN(IV), though the latter did show a slightly higher activity after 4 reuses with nitrate addition (Figure S2).

Activity of CAN (IV) and CAN(III) was shown to exhibit slight differences, where CAN(IV) was found to be more efficient at maintaining recyclability but the CAN(III) more efficient for the initial run. The increasing ionic charge might explain this, where increased Lewis acid capability allows CAN(IV) to act as a better catalyst. As for explaining the cases where CAN(III) is a better catalyst, further experiments were conducted. Michael additions with CAN were attempted with various amines to see how steric bulk affected the Michael addition and further probe differences between the two oxidation states; Figure S3 shows the dipropylamine (DPA, Figure S3A) and dibutylamine (DBA, Figure S3B). Interestingly, the difference between CAN(IV) and CAN(III) appears to increase with increasing steric bulk of the amine. Dicyclohexane (DCHA) and diisopropylamine (DIPA) were also tested however no reaction appeared to take place, most likely due to the much more bulky sterics of diisopropyl and cyclohexyl groups. Ce(III) is a bigger ion due to reduced orbital contraction and the increased relative size may have the effect of allowing increasing co-ordination with substrates. This together with one less nitrate group and the increased nucleophilicity of dibutylamine (and relatively small increase in sterics in comparison with the propyl group) might explain the increase rates of reaction seen with the large amine group and Ce(III).

Another consideration was that the Michael adduct, **1**, might be hindering the catalytic ability of CAN (both III and IV). Therefore, prior to the Michael addition reaction, CAN(IV) and CAN(III) were stirred for 2 h with a small amount of purified product **1**, this was then removed before performing the reaction as normal. The results are shown in Figure S4 (Figure S4B, shows a comparison without this extra pre-reaction step). Initial observations suggest that the addition of product **1** actually improves the reaction; however this is very unlikely to be the case. Incomplete removal of the addition product from the CAN increases the amount of addition product in the reaction, which invalidates the experiment. However, considering the increases in adduct yield as time passes, it is reasonable to conclude that the addition product had no effect, positive or negative, on the catalytic ability of CAN.

Interestingly, a consistent observation was that the CAN(IV) catalyst starts as an orange powder, but after the first reuse the color changes dramatically to give a brown sticky residue that adheres to the side of the reaction vessel. It was considered that this change in physical state might reduce available reactive surface area as well as the catalyst structure itself. In order to avoid agglomeration of the catalyst to a residue, CAN (IV) was physisorbed on to silica, with the aim of retaining small particle sizes with good accessibility. Two different loadings of CAN(IV) on silica were prepared to investigate the effect of loading on reuse and catalytic activity. Figure S5 shows a comparison of catalytic activity between the free and two different immobilized CAN(IV) catalysts and surprisingly, the same loss of activity after reuse is observed and appears to be independent of catalyst loading. Although catalytic activity appears not to be a result of surface area loss, the immobilized CAN(IV) was more easily recovered a reused and therefore this approach still offers some merit, despite not overcoming the catalyst deactivation.

### Alternative Catalysts to CAN for Aza-Michael Additions

**Figure 7:**
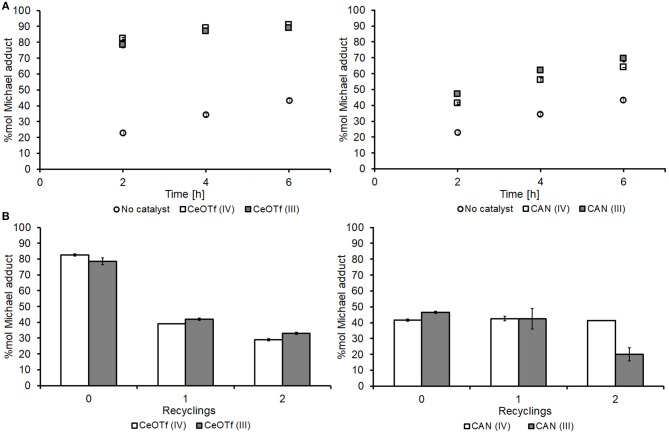
Comparison and recyclability of the CAN vs. the CeOTf catalysts. **(A)** Time course of the aza-Michael addition of DEA on DMI using the CeOTF (left) and the CAN (right) catalysts alongside no catalyst control; **(B)** recyclability of the CeOTf (left) and the CAN (right) catalysts. Spectra of the crude reaction mixture was recorded in CDCl_3_ after 2 h of reaction. All experiments were performed in duplicates and are shown in the figure as the average of two independent experiments ± the standard deviation. The catalysts were used as received (Sigma-Aldrich) without pre-treatment.

CANs deactivation issues led us to search for alternative catalysts for the reaction between DMI and DEA. Complexes containing trifluoromethanesulfonate (OTf) ligands are known to be useful in metal-centered catalysis due to their ability to make the central metal ion highly electrophilic, and further to this, there have been many literature reports of complexes containing OTf being recyclable as well as efficient for Michael additions (Kobayashi et al., 1992). Both Ce(IV) and Ce(III) OTf were tested and initially showed much more promising yields to Michael adduct **1**, being considerably higher than those achieved with the CAN catalyst (Figure 7A). Unfortunately, Figure 7B shows that the catalytic ability of CeOTfs reduces significantly upon reuse, achieving similar conversions to the non-catalyzed background syntheses, suggesting almost total deactivation. A comparison between the two oxidation states for the OTf salts showed that the 4+ state is a better catalyst until it is consumed or made ineffective, thereafter the 3+ state dominants, contrary to the trend observed for the CAN catalysts. It is possible that in CeOTf, Lewis acidity is the primary mechanism of catalysis (more so than the CAN) in which case a more electrophilic Ce(IV) is a better Lewis acid.

This data led us to seek an alternative to lanthanide catalysts. A further screen of the literature suggested molecular iodine (I_2_) as a promising candidate since it is reported to catalyze the addition of various aliphatic Michael acceptors and donors at high yields (>80%) (Borah et al., 2010). The amount of catalyst (based on relative %mol of I_2_ to Michael acceptor) was screened at 1.5, 5, and 12.5%mol (entries 2–4, Table 1). Molecular iodine gave impressive yields (76–90%) for all three loadings, giving considerably higher yields than using no catalyst (entry 1, 26–28%), and producing limited amounts of the unwanted DMMes isomer. However, the molecular I_2_ dissolved entirely in the excess of DEA, making it unrecoverable. Saikia et al. previously showed that I_2_ can be supported onto alumina and used for other aza-Michael additions. This approach was therefore considered and further investigated (Saikia et al., 2009). Acidic alumina (i.e., without I_2_), used as received, was found to have no catalytic ability (entry 5, Table 1), giving similar results to the no catalyst system (entry 1) with the exception of a slight increase in the isomerization of DMI to DMMes. The low catalytic ability of acidic alumina differs from observations of Bosica and Abdilla who found it to be suitable catalyst for aza-Michael additions of aromatic amines (Bosica and Abdilla, 2016). However, acidic alumina was found to be a suitable support for supporting I_2_. Using the preparation method given by Deka and Sarma a free-flowing heterogeneous catalyst was prepared and found to give reasonable yields of adduct even upon recovery (*via* vacuum-assisted Buchner filtration and air drying) an subsequent reuse (entry 6, Table 1; Deka and Sarma, 2001). Nitrogen porosimetry (Figure S6) showed the BET surface area of the acidic alumina (120 m^2^ g^−1^) decreased slightly upon supporting 0.1 mmol g^−1^ fresh I_2_ (110 m^2^ g^−1^), and decreased further for the recovered catalyst (86 m^2^ g^−1^). Pore size distribution would suggest this reduction in surface area was a result of partial blocking of the mesopores (Figure S7), though despite this reduction in surface area the 0.1 mmol g^−1^ I_2_ on acidic alumina maintained its catalytic ability upon reuse (entry 6, Table 1). Thermogravimetric analysis (TGA) corresponds to the porosimetry trends, showing the mass loss up to 625°C for the acidic alumina support (~5% loss, Figure S8A) increases slightly to 7–8% loss following 0.1 mmol g^−1^ loading of I_2_ (Figure S8B). Of note is that the loss of I_2_ does not appear to give a specific narrow temperature range for desorption, but instead seems to occur over a broad temperature range. The recovered catalyst's TGA trace (Figure S8C) shows an additional mass loss over the 30–220°C range, this attributed both to some residual amine (30–80°C, diethylamine b.p. is 55.5°C) and trapped itaconate, mesaconate, and adduct (100–220°C). The residual organics observed by TGA likely also caused the surface area and pore size reduction seen from porosimetry.

**Table 1:** Catalyst screen for the solvent-less reaction of DEA with DMI after 2 h.

**Entry**	**Catalyst**	**1st or reuse**	**%mol DMI**	**%mol DMMes**	**%mol Michael adduct 1**
1	No Catalyst	1st use	50	22	28
		Repeat	51	23	26
2	Iodine (I_2_)^a^ (1.5%mol)	1st use	13	20	67
		Re-use	Homogeneous—not recoverable and reusable		
3	Iodine (I_2_)^a^ (5%mol)	1st use	4	16	80
		Re-use	Homogeneous—not recoverable and reusable		
4	Iodine (I_2_)^a^ (12.5%mol)	1st use	9	1	90
		Re-use	Homogeneous—not recoverable and reusable		
5	Acidic alumina^b^	1st use	44	27	29
		Re-use	47	27	26
6	I_2_ on acidic alumina (0.1 mmol g^−1^ I_2_ loading)	1st use	6	21	73
		Re-use	4	22	74
7	I_2_ on neutral alumina (0.1 mmol g^−1^ I_2_ loading)	1st use	20	27	53
		Re-use	14	28	58
8	I_2_ on basic alumina (0.1 mmol g^−1^ I_2_ loading)	1st use	5	25	70
		Re-use	7	24	69
9	K60 silica^c^	1st use	41	29	30
		Re-use	35	30	35
10	I_2_ on K60 silica (0.1 mmol g^−1^ I_2_ loading)	1st use	22	35	43
		Re-use	14	31	55
11	Amberlyst-15 (0.25 g)	1st use	40	29	31
		Re-use	26	34	40

*2.5 mmol DMI, 20 mmol DEA, 5%mol (w.r.t. I_2_ per mole of DMI = 1.28 g of standard catalyst) catalyst unless otherwise stated, stirred for 2 h at room temperature, analysis by NMR spectroscopy (CDCl_3_ solvent)*.

a *Molecular iodine had no Run 2 as it was not recoverable from the reaction mixture*.

b *Mass of acidic alumina used equals that used for I_2_ on alumina catalyst (1.25 g)*.

c *Mass of K60 silica used equals that used for I_2_ on K60 silica (1.25 g)*.

Equivalent supported forms of 0.1 mmol g^−1^ I_2_ on neutral (entry 7, Table 1) and basic (entry 8, Table 1) alumina were also prepared and trialed for the addition of DEA to DMI, but the original system on acid alumina was found to remain superior. 0.1 mmol g^−1^ I_2_ on K60 silica (I_2_-SiO_2_) was also assessed as a potential heterogeneous form of iodine (entry 10, Table 1), though this proved less efficient than the alumina supported systems. The I_2_-SiO_2_ seemingly promoted undesirable isomerization to a greater extent (>30%) compared to the other catalysts, an observation similarly observed for K60 silica without iodine (entry 9, Table 1). Amberlyst-15, an acidic heterogeneous resin, has also been reported to catalyze aza-Michael additions though for our substrate was found to have limited activity (entry 11, Table 1; Das and Chowdhury, 2007).

### Extended Re-use Study for I_2_ on Acidic Alumina

**Figure 8:**
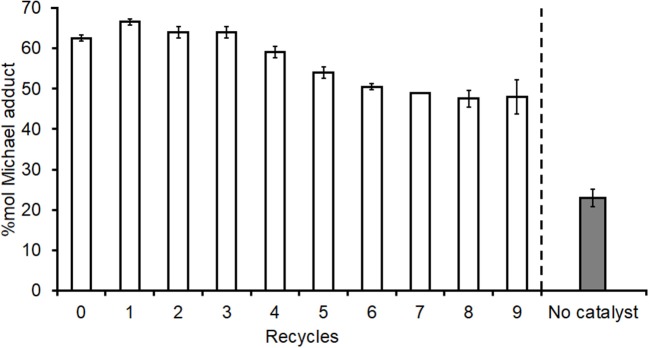
Extended re-use study of I_2_ on acidic alumina catalyst for addition of diethyl amine onto dimethyl itaconate. 2.5 mmol DMI, 20 mmol DEA, 1.28 g of 0.1 mmol g^−1^ I_2_ on Al_2_O_3_ (standard loading) catalyst, stirred for 2 h at room temperature, reaction mixture filtered then filtrate analyzed by NMR spectroscopy (CDCl_3_ solvent) while catalyst was recovered and reused with fresh reactants. Following filtration the catalyst was air-dried overnight prior to reuse between each run. All experiments were performed in duplicates and are shown in the figure as the average of two independent experiments ± the standard deviation.

**Figure 9:**
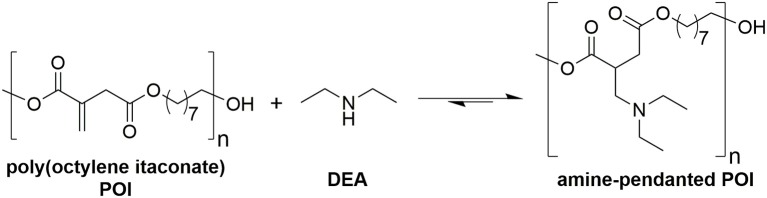
Reaction scheme for addition of diethyl amine (DEA) pendant onto poly(octylene itaconate) (POI).

An extended recyclability study to 10 full cycles was carried out using the standard loading 0.1 mmol g^−1^ I_2_ on acidic alumina catalyst (Figure 8) for the addition of DEA onto DMI. The first use and subsequent first four recycles retain good catalytic activity, whilst a slight reduction in activity was observed after the 5th recycle. Despite yields to adduct **1** dropping to ~48% for the last three uses, this remained considerably higher than with no catalyst at all (26–28% entry 1, Table 1, and Figure 9) or when acidic alumina without I_2_ was used (29%, entry 5, Table 1). The catalyzed experiment started with 1.28 g of catalyst but after the 10 full cycles 1.06 and 0.97 g (duplicate) of final catalyst was recovered. As such the final runs had catalyst loadings reduced to 76–83% of the original and this mass loss may have contributed to some of the reduced adduct yield observed over the extended reuse study. TGA analysis of the fresh (B, Figure S9) and recovered catalyst after 10 uses (C, Figure S9) suggest the latter contains 10–11% additional mass contributed by residual organics, this likely blocking some active sites and further reducing catalytic activity as observed in Figure 8. Nevertheless, I_2_-Al_2_O_3_ was proven to be a more appropriate heterogeneous and recyclable catalyst for the aza-Michael addition compared to the lanthanide catalysts trialed above.

### Effects of I_2_ Loading on I_2_ on Acidic Alumina Catalyst

Increasing I_2_ loading on acidic alumina was investigated by multiplying the amounts of I_2_ deposited onto the same amounts of catalyst (2x, 3x, and 4x). For their use in the aza-Michael addition the mass of each catalyst was concurrently reduced so as to ensure the same amount of I_2_ (12.5%mol of I_2_ with respect to DMI) was present in each run despite less acidic alumina support as loading increased. Results in Table 2 show that the standard loading (1x = 0.1 mmol g^−1^ I_2_-Al_2_O_3_) and double loading (2x = 0.2 mmol g^−1^ I_2_-Al_2_O_3_) gave very similar results for 1st use and reuse, suggesting these suffer little from I_2_ leaching. Increasing to 3x and 4x loadings both gave marginally higher yields of adduct for the first use, and the 4x loading suffered from a noticeable drop in yield upon reuse suggesting some leaching of I_2_ occurred during the first run. Free, homogenous I_2_ is extremely active as a catalyst for this reaction (see entries 2–4, Table 1, entry 4 being the direct comparison with 12.5%mol I_2_ relative to DMI) and therefore leaching for the 4x, and possibly 3x, is likely responsible for the 1st use giving a greater yield. Based on this data it was concluded that the 2x loading (0.2 mmol g^−1^ I_2_-Al_2_O_3_) was the optimum catalyst and this was used for a study into the catalyzed addition of DEA onto an itaconate polyester.

**Table 2:** Effect of increased I_2_ loading on acidic alumina for the Michael addition between DEA and DMI.

**Entry**	**I_**2**_ loading on acidic alumina**	**1st or reuse**	**%mol DMI**	**%mol DMMes**	**%mol Michael adduct 1**
1	1x (standard)	1st use	3	22	75
		Re-use	4	18	78
2	2x	1st use	2	22	76
		Re-use	3	20	77
3	3x	1st use	2	17	81
		Re-use	2	18	80
4	4x	1st use	1	15	84
		Re-use	4	20	76

*2.5 mmol DMI, 20 mmol DEA, 12.5%mol I_2_ catalyst relative to DMI (3.2 g 1x loading, 1.6 g, 0.8 g and 0.67 g for 2x, 3x, and 4x loading, respectively), stirred for 2 h at room temperature, reaction mixture filtered then filtrate analyzed by ^1^H-NMR spectroscopy (CDCl_3_ solvent)*.

### Use of I_2_ on Acid Alumina for Catalyzing the Aza-Michael Addition Onto Itaconate Polyesters

**Figure 10:**
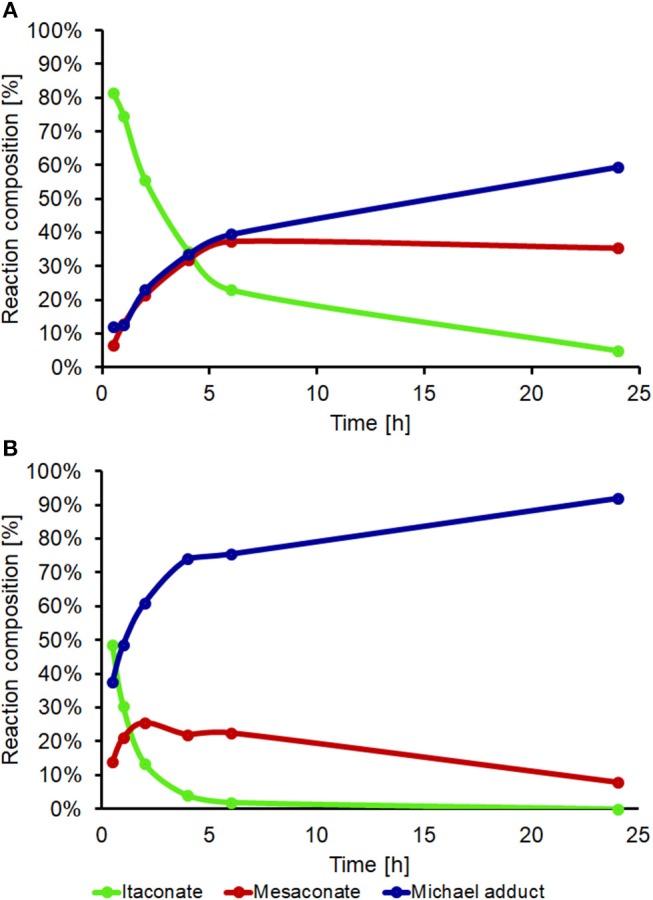
Aza-Michael addition of DEA on poly(1,8-octylene itaconate) (POI). **(A)** Non-catalyzed reaction and **(B)** I_2_ on Al_2_O_3_ as the catalyst. 2.5 mmol of POI (0.6 g, based on constitutional repeat unit of 240.29 g mol^−1^), 20 mmol DEA, 0.64 g of 0.2 mmol g^−1^ I_2_ on Al_2_O_3_ catalyst added to **(B)**, room temperature, aliquots analyzed by ^1^H-NMR spectroscopy (CDCl_3_ solvent) at 0.5, 1, 2, 4, 6, and 24 h.

DEA addition onto poly(1,8-octylene itaconate) (POI, Figure 9) was selected as an example reaction to study whether iodine on acidic alumina remained catalytic for reactions involving polymer substrates where viscosity is significantly increased and mass transfer thus reduced. The 2x loaded catalyst (0.2 mmol g^−1^ I_2_ on Al_2_O_3_) was selected from the screen above. Maximizing the loading of I_2_ would reduce the mass transfer limitations by lowering the overall quantity of required solid. A comparison against the uncatalysed system was made, and in both instances an 8:1 molar ratio of DEA to itaconate units was used, thus the excess DEA also acting as a solvent. The POI polyester was prepared using a previously reported enzymatic catalyzed polymerization method, this to minimize isomerization caused during polymerization. The method used for determining the extent of addition and isomerization via ^1^H-NMR spectroscopy was modified from the model system (Figure 5) as a result of some peak overlap and loss of resolution (Figure S10 for POI, Figure S11 following addition of DEA to the polymer). The reference peak changed from the methyl ester group to the combined signals around 4.2 ppm set to an integral of 4 (CH_2_s of the 1,8-octanediol nearest to the esters). The amount of addition was determined based on loss of alkene proton signals (5.5–6.8 ppm), whilst the amount of isomerization to mesaconate was determined by comparing signal integrals of the alkene protons of mesaconate (6.8 ppm, 1H) and itaconate (5.7 and 6.3 ppm, 1H each). A dramatic increase in rate of formation of aza-Michael adduct pendanted polyester was seen when using the I_2_ on acidic alumina catalyst, monitored over a 24 h period (Figure 10). The catalyzed reaction had a 92% conversion rate to the Michael adduct polyester after 24 h, with only 60% seen for the conventional uncatalysed system. The catalyst also attained adduct yields of >70% after just 4 h, and this with a concurrent significant reduction in extent of isomerization to mesaconate. The rapid reduction in the amount of itaconate units, with nearly complete conversion after 6 h, further demonstrates this catalyst to be very effective in promoting aza-Michael additions onto itaconate whilst not promoting undesired regio-isomerization. It was noted that the catalyzed reaction resulted in a polymer of darker orange coloration compared to that of no catalyst. Thermogravimetric analysis (Figure S12) of the fresh and recovered catalyst was thus used to ascertain if an appreciable quantity of I_2_ was lost from the surface of the catalyst during the course of the reaction. The fresh 0.1 mmol g^−1^ I_2_ on Al_2_O_3_ catalyst showed a prolonged gradual mass loss over the full TGA range (30–625°C) representing ~7–8%. Complete loss of all I_2_ from this sample should have resulted in a mass loss of just 2.5%, therefore it was hypothesized that water adsorbed onto the Al_2_O_3_ was also contributing to the observed mass loss. This was confirmed by comparison to the acidic alumina as received (Sigma-Aldrich), where a gradual mass loss of ~5% is indeed also observed (Figure S8A). The recovered spent catalyst following the addition of DEA to DMI also shows this prolonged mass lost but has an additional mass loss at 190–270°C. This new mass loss event matches the previously reported temperature for retro-aza-Michael addition of the DEA unit from the polymer, suggesting residual polymer is contained on or within the catalyst (Pellis et al., 2019). The gradual mass loss associated to I_2_ and H_2_O release matches roughly with that for the fresh catalyst suggesting leaching of I_2_ was only a minimal cause of the observed coloration, and further supports the sound reusability of this catalyst.

## Conclusion

Although a versatile and useful reaction for the derivatization of α,β-unsaturation carbonyls, aza-Michael additions typically require long reaction times of several days. This is particularly problematic for the addition of amine pendants onto bio-based itaconate polyesters as undesired regioisomerization results in formation of mesaconate units which significantly reduce the rate of aza-Michael addition. A screen of various heterogeneous catalysts using the model reaction of addition of diethylamine onto dimethylitaconate found that I_2_ supported onto acidic alumina produced an effective, recoverable and reusable catalyst for this aza-Michael addition. Although initially promising it was eventually concluded that various catalysts based on a cerium metal center were ineffective due to rapid deactivation after their first use. Despite extensive studies we were unable to fully ascertain the cause of this deactivation and hence sought an alternative. I_2_ supported on acidic alumina demonstrated far better reusability, with an extended reuse study still showing significant catalytic activity remained. The best supported iodine catalyst gave yields of aza-Michael adduct of >70% after 2 h, even after reuse, while the equivalent non-catalyzed reaction had yields of adduct of 26–28%. A screen of I_2_ loading found that catalysts of ≤ 0.2 mmol g^−1^ I_2_ on alumina maintained their original efficiency upon reuse, while higher loadings of I_2_ saw a drop that was likely caused by leaching of iodine into the reaction media. The optimum catalyst was subsequently used for the addition of diethylamine onto poly(1,8-octylene itaconate), this unsaturated polyester prepared via enzymatic polycondenzation. Use of the catalyst for post-polymerization modification showed that >90% amine pendant addition was possible after 24 h, this was far greater than the non-catalyzed equivalent with just 60% adduct and extensive undesired mesaconate units remaining. More impressively the catalytic system showed >70% addition after just 4 h, the non-catalyzed equivalent reached than half this over the same period. This study thus concludes that I_2_ supported on acidic alumina is a very effective catalyst for aza-Michael additions on bio-based itaconate polyesters, and holds much promise in considerably reducing the lengthy times typically used for these reactions.

## Data Availability

The raw data generated for the iodine on acidic alumina study and used to confirm the results can be found via doi: 10.15124/0bf5104e-70aa-4bb8-958b-a7501dcd2b48. Raw data for the cerium catalyst section of the study is available from the authors upon request.

## Author Contributions

TF developed the initial concept. TF, AP, and JC supervised the study. JC prepared immobilized CAN samples. AP prepared poly(1,8-octylene itaconate) and carried out TGA analysis. P-AH carried out the Ce catalyzed reactions, OM carried out the I_2_ catalyzed reactions (DMI and polymer), and porosimetry analysis of the catalysts. TF and AP led preparation of the manuscript but were assisted by all the authors.

### Conflict of Interest Statement

The authors declare that the research was conducted in the absence of any commercial or financial relationships that could be construed as a potential conflict of interest.
