# Supplementary figures and images for: Improving the Post-polymerization Modification of Bio-Based Itaconate Unsaturated Polyesters: Catalyzing Aza-Michael Additions With Reusable Iodine on Acidic Alumina
Source: Front Chem. 2019 Jul 15;7:501. doi: 10.3389/fchem.2019.00501 (PMC6644777; doi:10.3389/fchem.2019.00501)

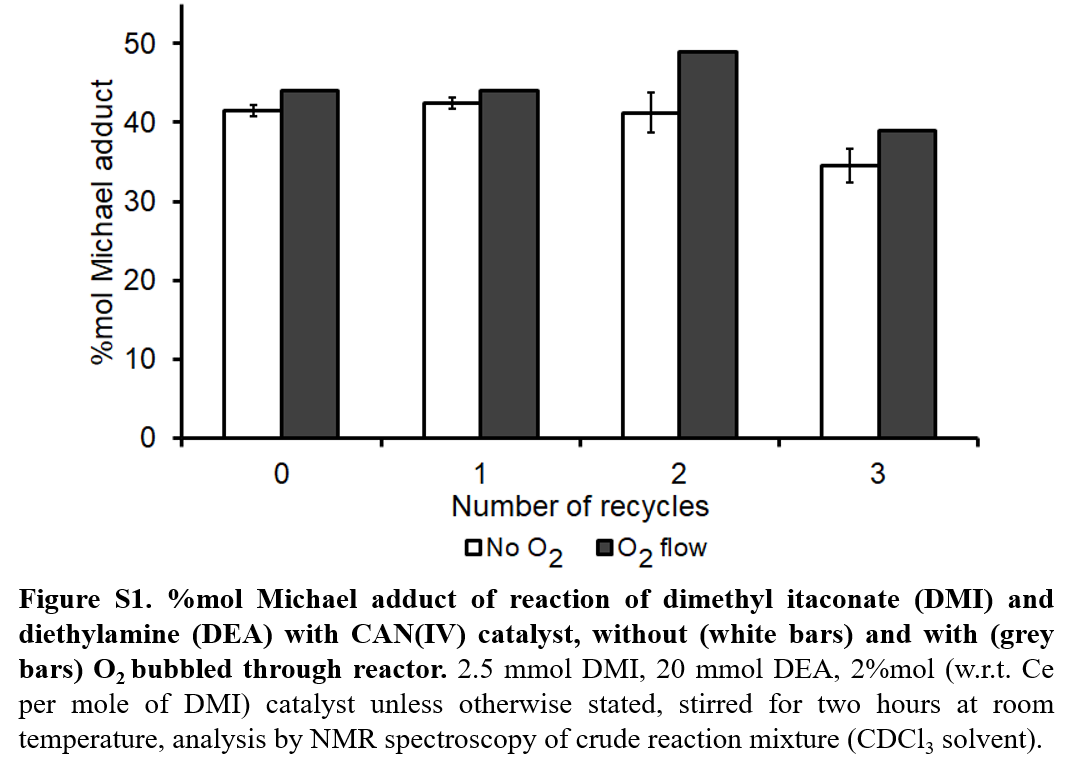

Supplement: Supplementary file 1 [file Image_1.TIF]

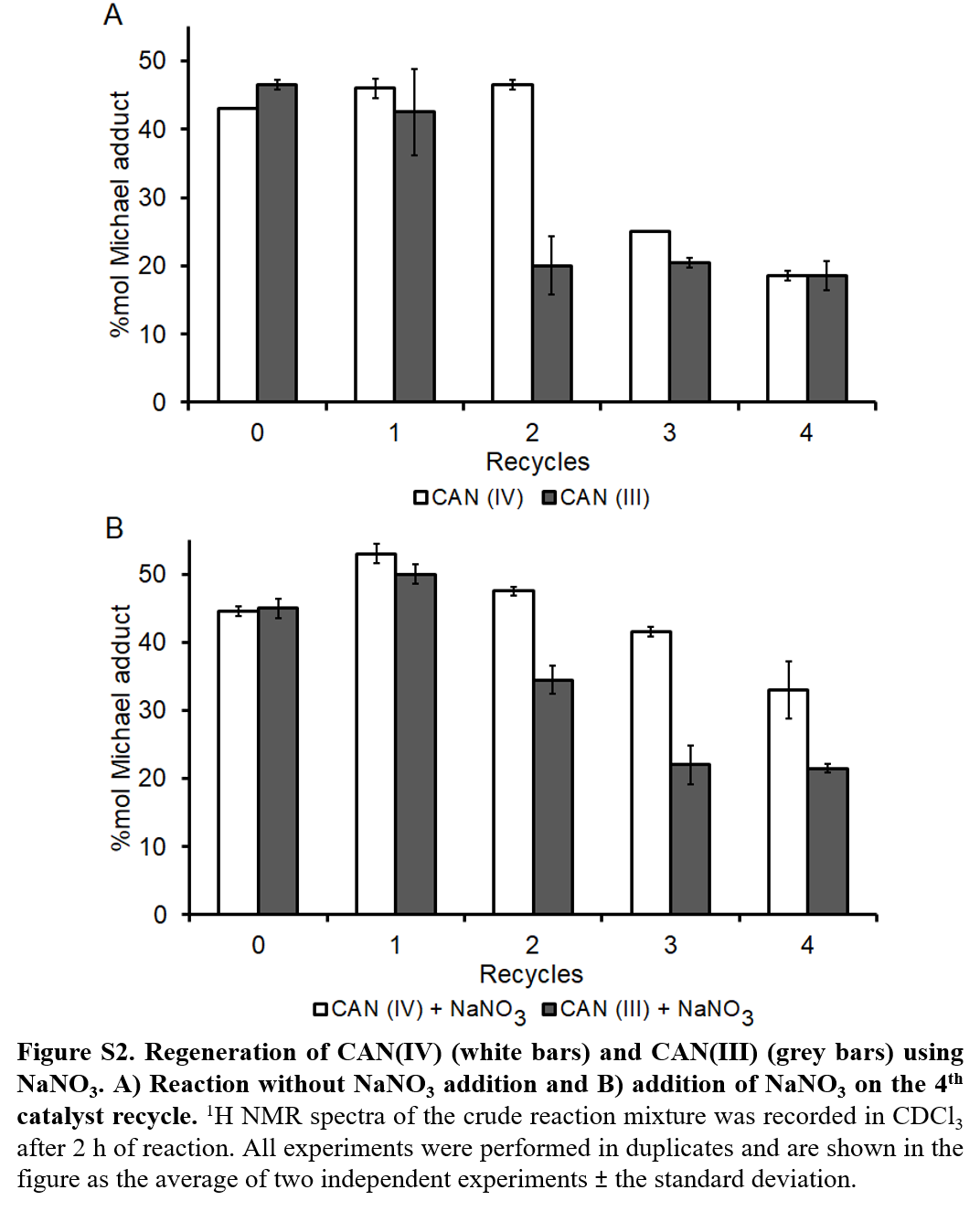

Supplement: Supplementary file 2 [file Image_2.TIF]

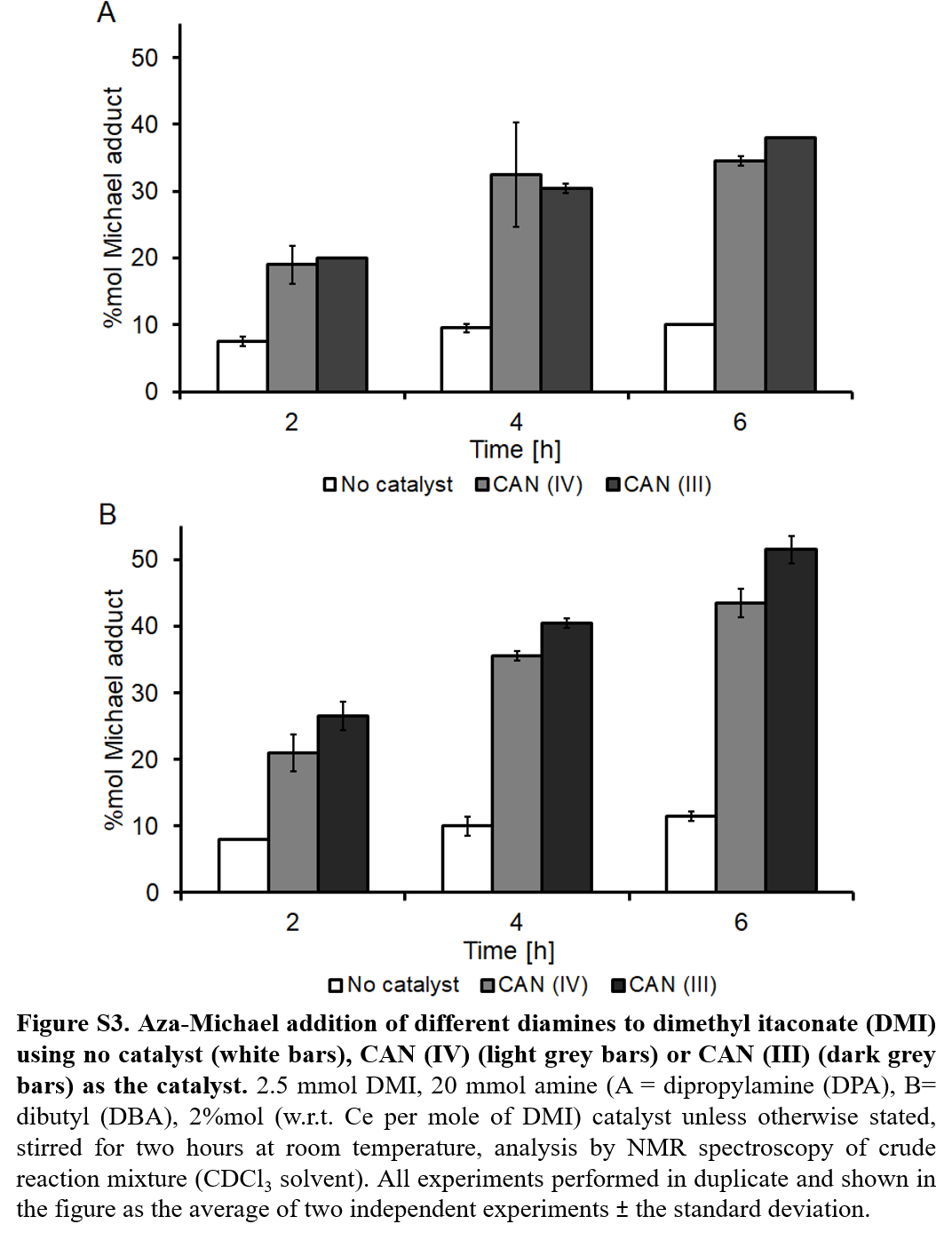

Supplement: Supplementary file 3 [file Image_3.tif]

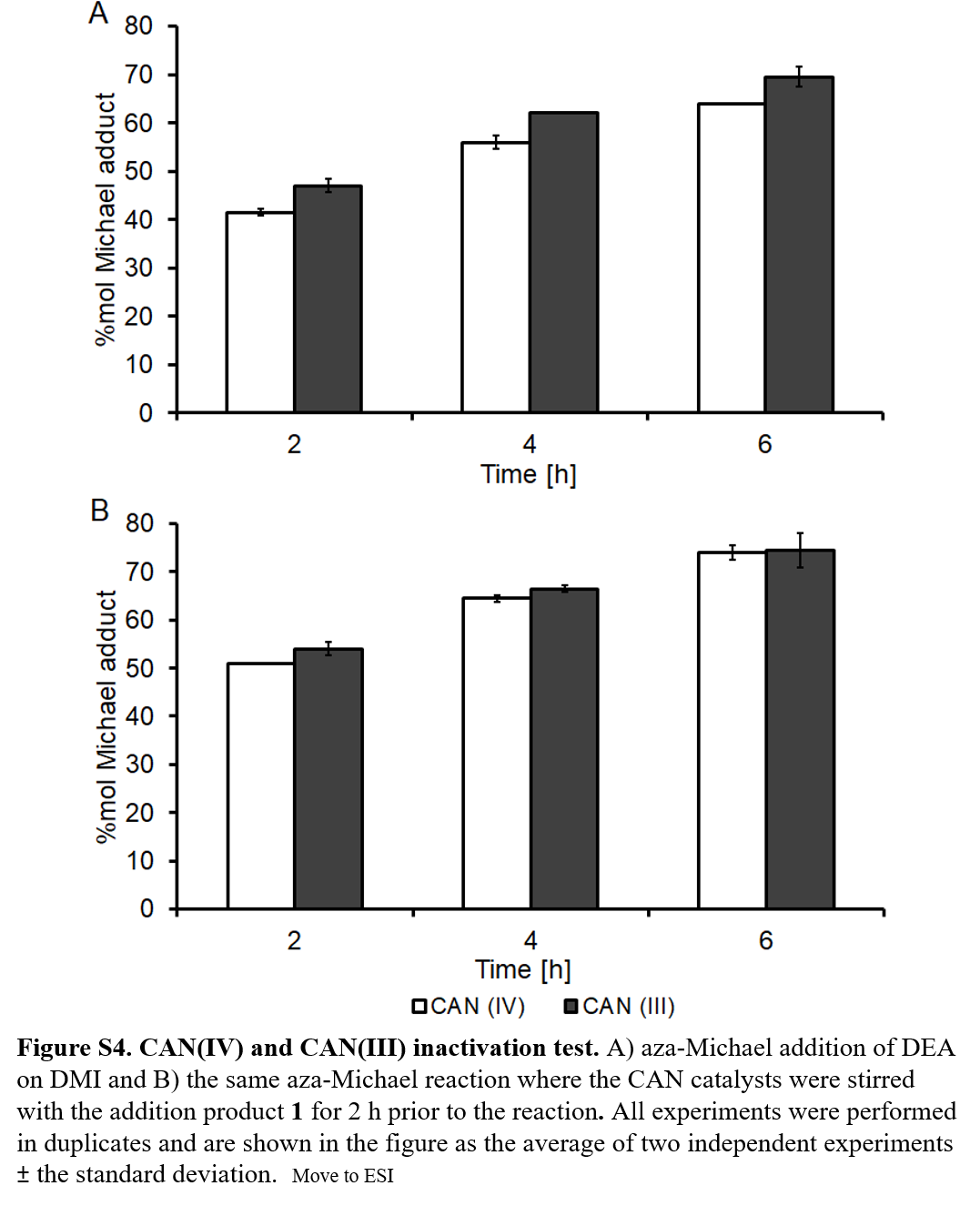

Supplement: Supplementary file 4 [file Image_4.TIF]

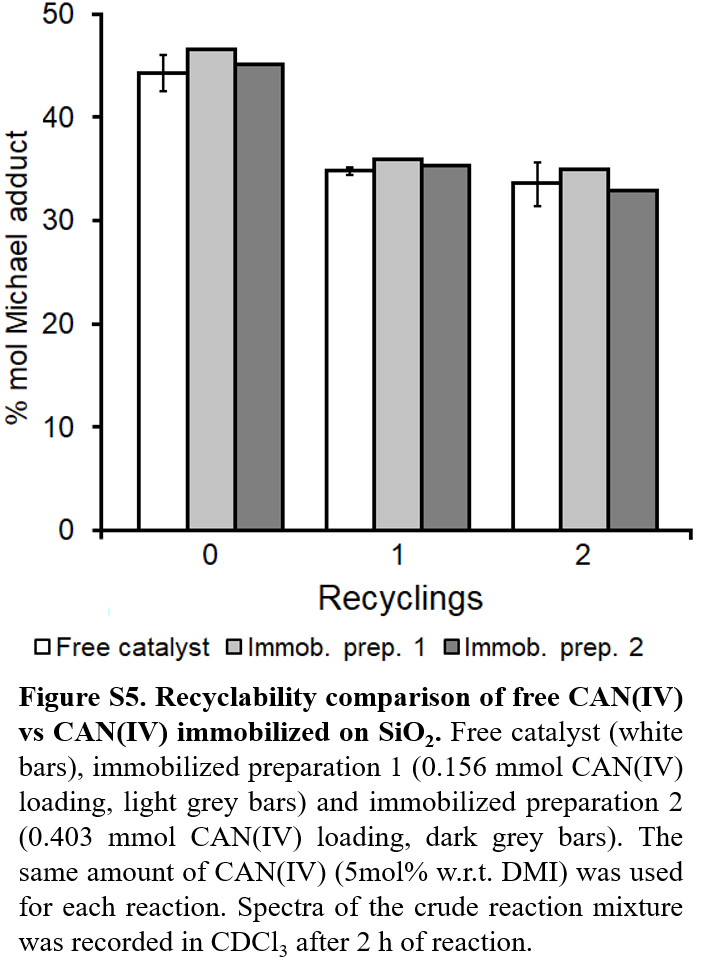

Supplement: Supplementary file 5 [file Image_5.TIF]

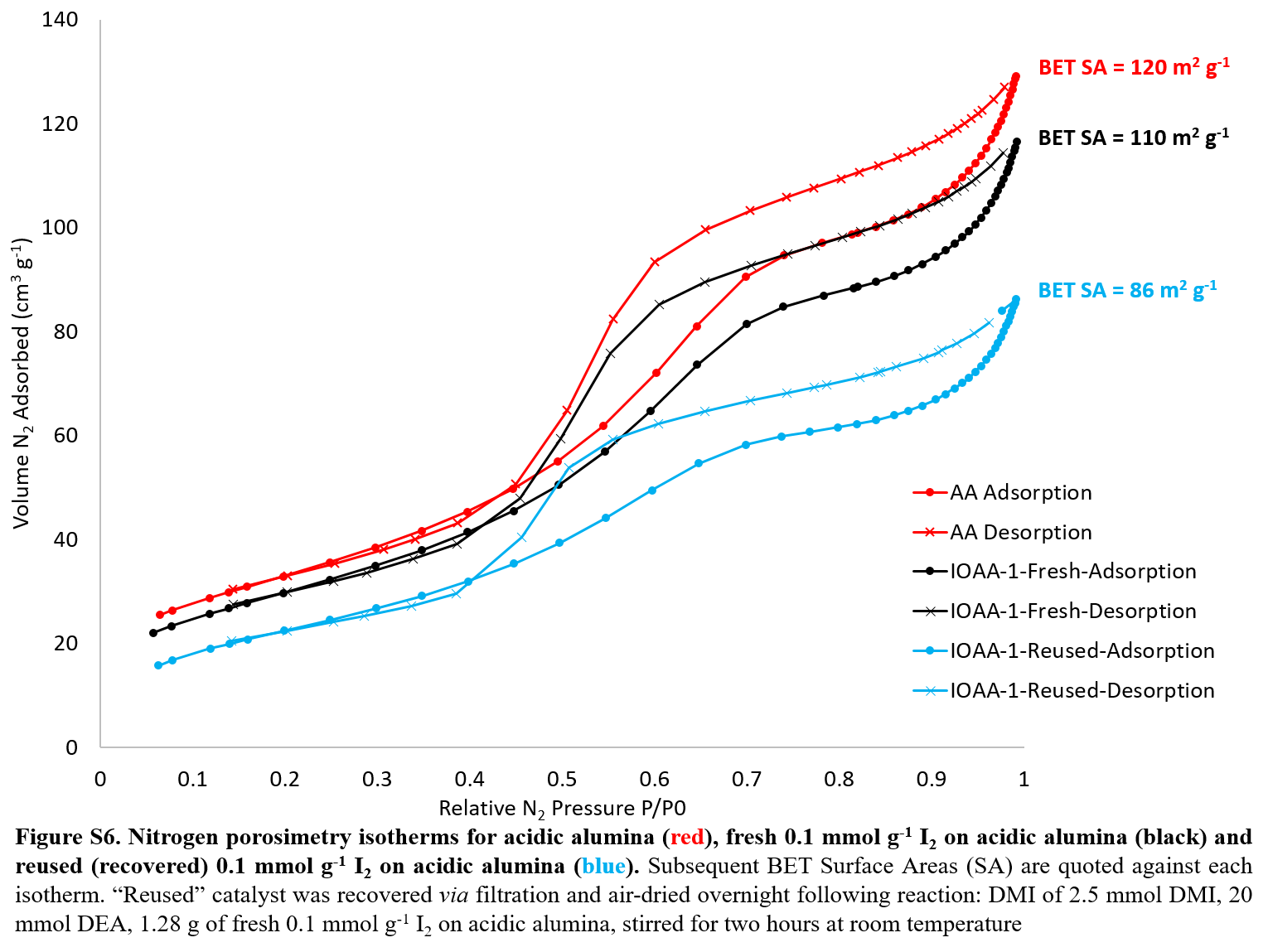

Supplement: Supplementary file 6 [file Image_6.tif]

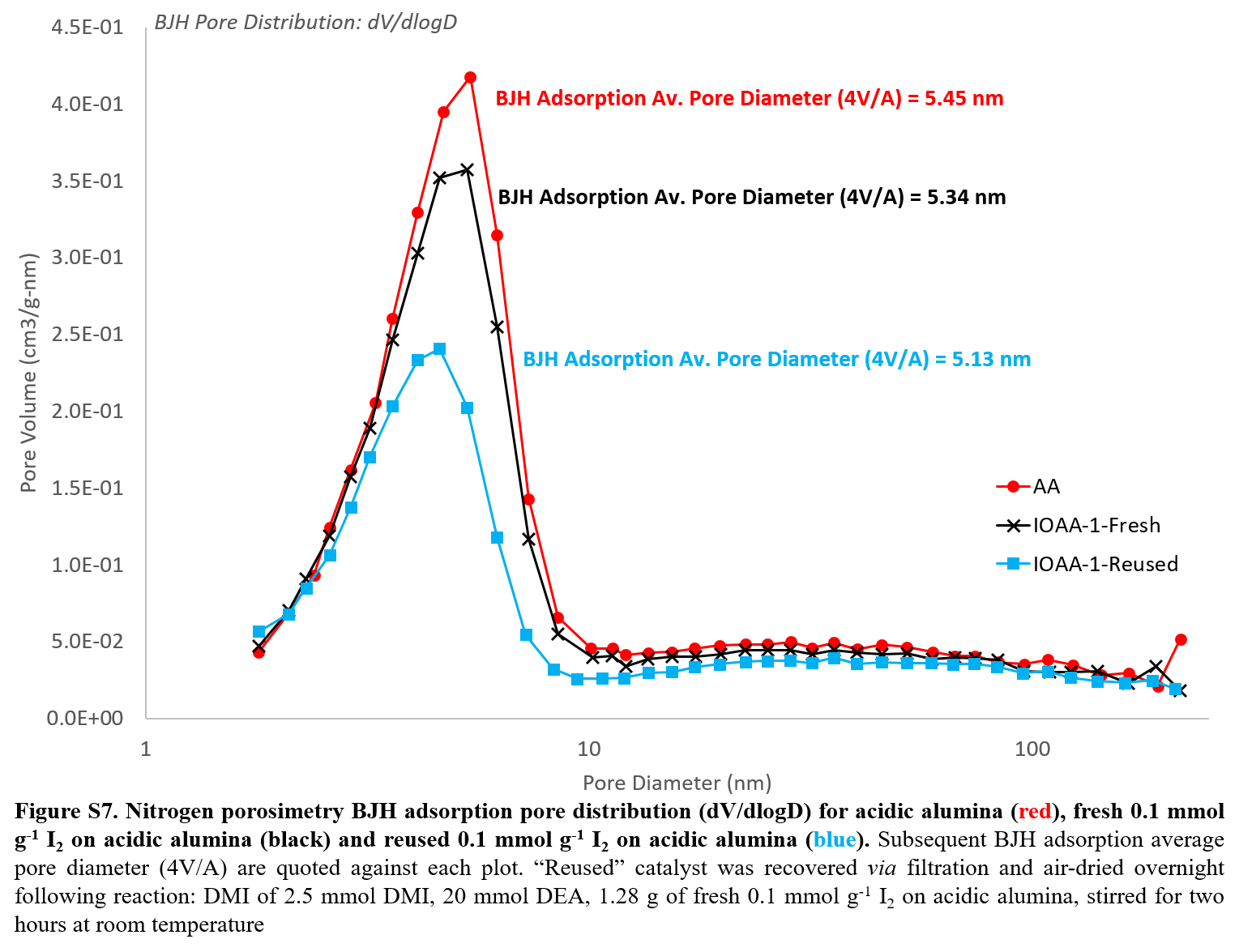

Supplement: Supplementary file 7 [file Image_7.TIF]

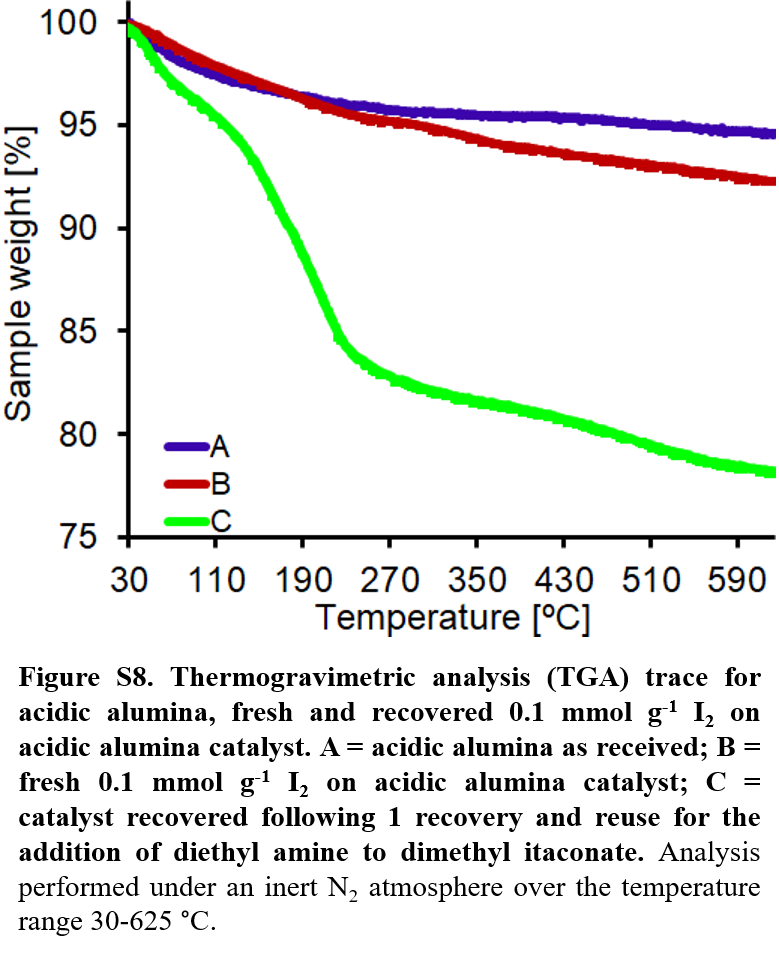

Supplement: Supplementary file 8 [file Image_8.TIF]

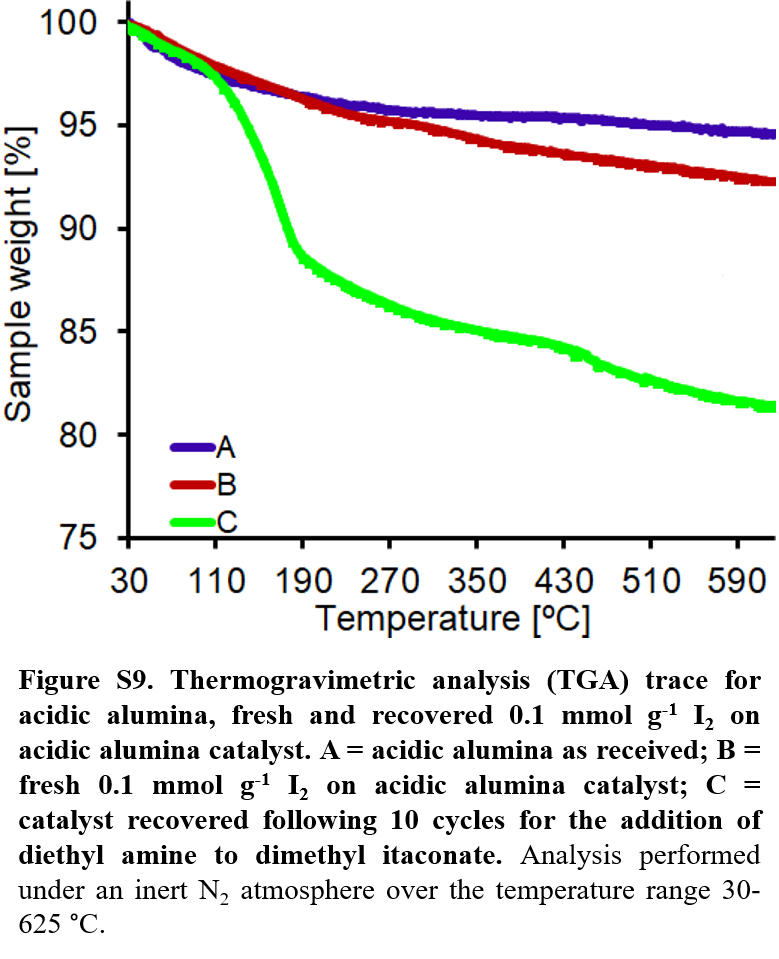

Supplement: Supplementary file 9 [file Image_9.TIF]

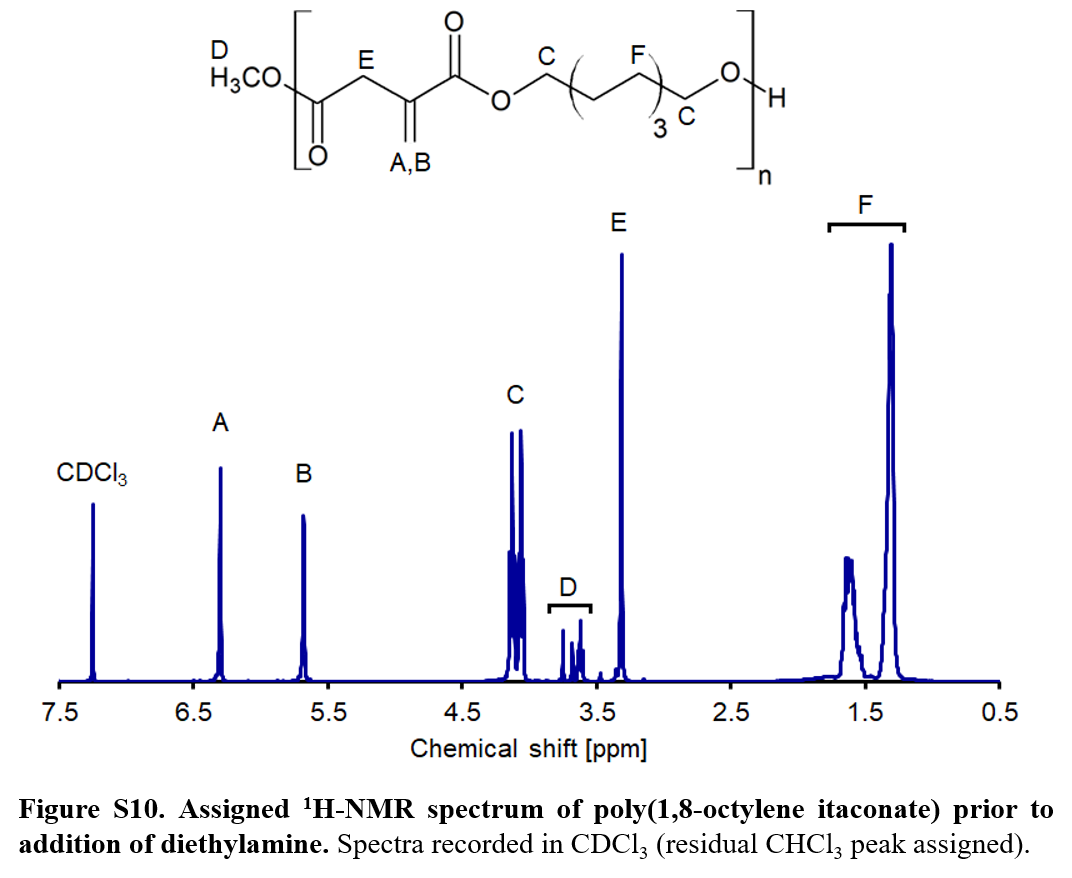

Supplement: Supplementary file 10 [file Image_10.TIF]

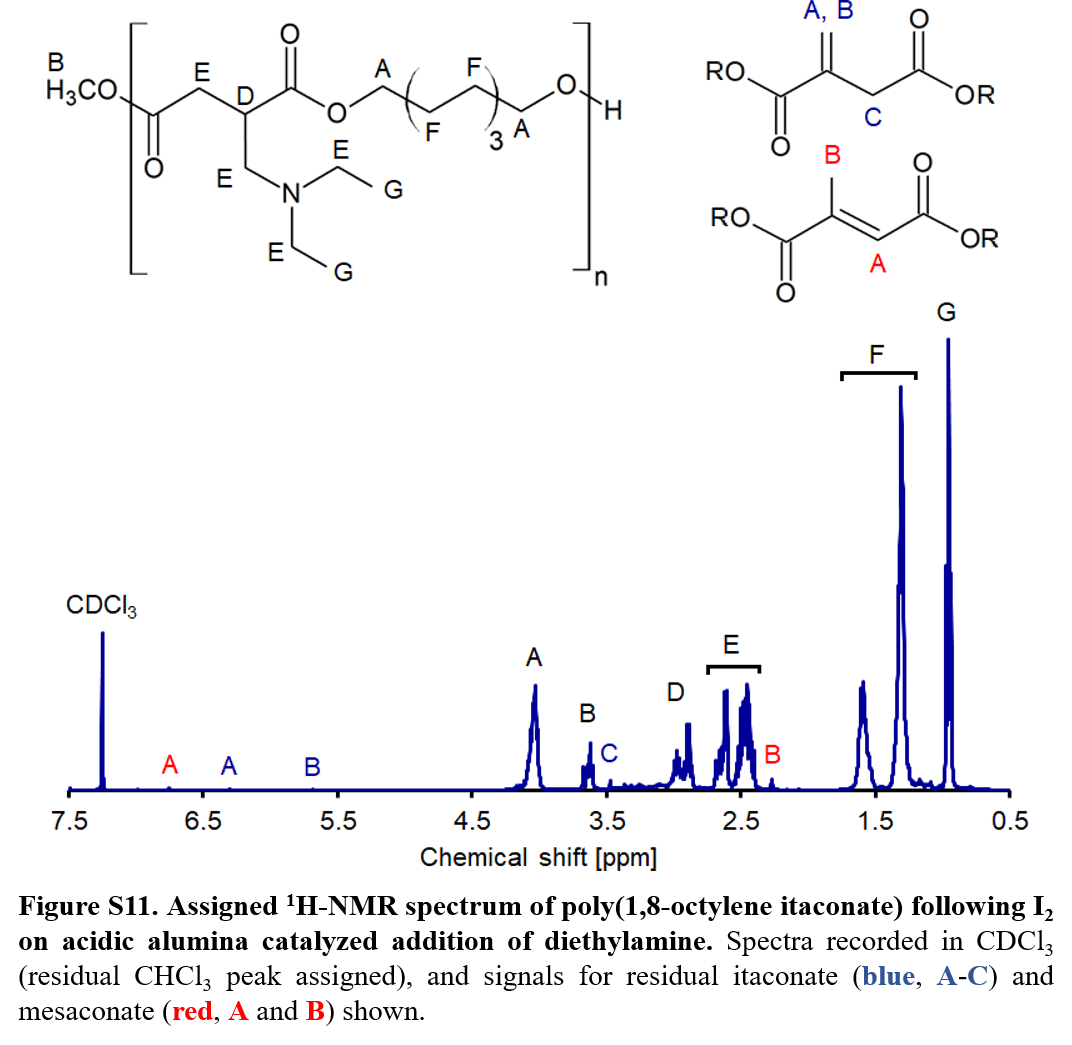

Supplement: Supplementary file 11 [file Image_11.TIF]

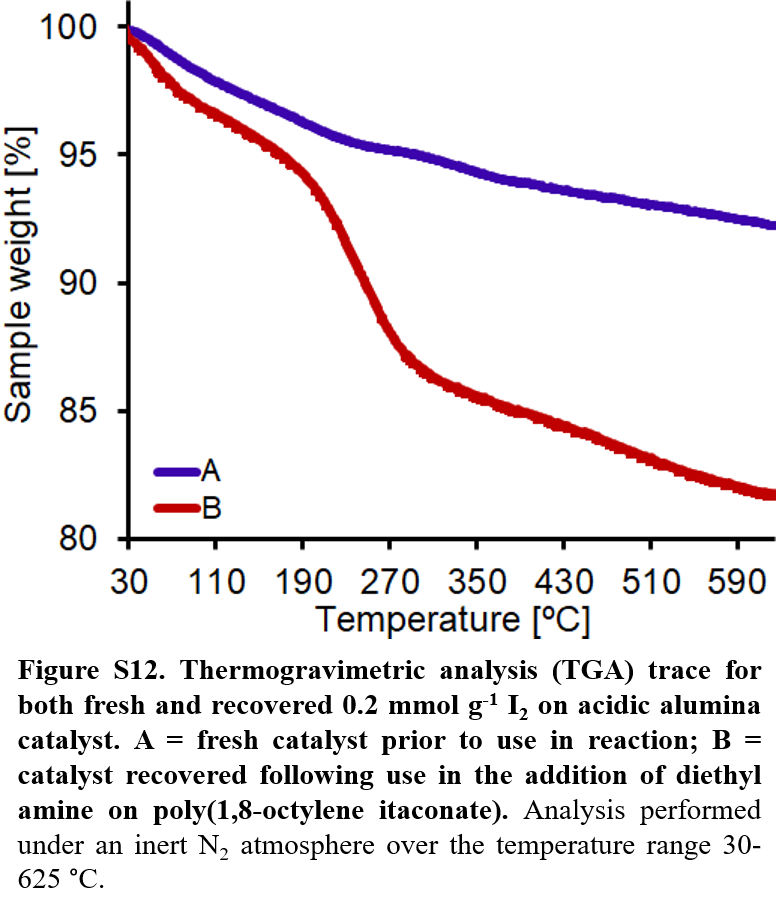

Supplement: Supplementary file 12 [file Image_12.TIF]
